# Interplay between lncRNA/miRNA and Wnt/ß-catenin signaling in brain cancer tumorigenesis

**DOI:** 10.17179/excli2023-6490

**Published:** 2023-11-28

**Authors:** Farzad Rahmani, Abdulridha Mohammed Al-Asady, Reyhane Hanaie, Mehrdad Zandigohar, Homeira Faridnejad, Mahya Payazdan, Pegah Safavi, Mikhail Ryzhikov, Seyed Mahdi Hassanian

**Affiliations:** 1 Kashmar School of Medical Sciences, Mashhad University of Medical Sciences, Mashhad, Iran; 2 Metabolic Syndrome Research Center, Mashhad University of Medical Sciences, Mashhad, Iran; 3 Basic Medical Sciences Institute, Mashhad University of Medical Sciences, Mashhad, Iran; 4 Department of Medical Sciences, Faculty of Nursing, University of Warith Al-Anbiyaa, Iraq; 5 Department of Pharmacology, Faculty of Medicine, Mashhad University of Medical Sciences, Mashhad, Iran; 6 Department of Biomedical Engineering, University of Illinois at Chicago, Chicago, IL, 60612, USA; 7 Department of Biophysics, East Carolina University, Greenville, NC, USA; 8 Department of Biology, Faculty of Sciences, Shahid Chamran University of Ahvaz, Ahvaz, Iran; 9 Department of Medical Radiation, Science and Research Branch, Islamic Azad University, Tehran, Iran; 10 Saint Louis University, School of Medicine, Saint Louis, MO, USA

**Keywords:** brain cancers, lncRNA, miRNA, Wnt/beta-catenin

## Abstract

Brain cancers are among the most aggressive malignancies with high mortality and morbidity worldwide. The pathogenesis of brain cancers is a very complicated process involving various genetic mutations affecting several oncogenic signaling pathways like Wnt/β-catenin axis. Uncontrolled activation of this oncogenic signaling is associated with decreased survival rate and poor prognosis in cancer patients. Long non-coding RNAs (lncRNAs) and microRNAs (miRNAs) were shown to play important roles in regulating cell proliferation, differentiation, and apoptosis by regulating the expression of their target genes. Aberrant expression of these non-coding RNAs (ncRNAs) was reported in many human cancers, including glioblastoma, medulloblastoma, meningioma, and pituitary adenoma. Multiple lncRNAs were shown to participate in brain tumor pathogenesis by targeting Wnt signaling regulatory miRNAs. SNHG7/miR-5095, PCAT6/miR-139-3p, SNHG6/miR-944, SNHG1/ miR-556‐5p, SNHG17/ miR-506-3p, LINC00702/miR-4652-3p, DLGAP1-AS1/miR-515-5p, HOTAIR/miR-1, HOTAIR/miR-206, CRNDE/miR-29c-3p, AGAP2-AS1/ miR-15a/b-5p, CLRN1-AS1/miR-217, MEG3/miR-23b-3p, and GAS5/miR-27a-5p are identified lncRNA/miRNA pairs that are involved in this process. Therefore, recognition of the expression profile and regulatory role of ncRNAs on the Wnt signaling may offer a novel approach to the diagnosis, prognosis, and treatment of human cancers. This review summarizes previous data on the modulatory role of lncRNAs/miRNAs on the Wnt/β-catenin pathway implicated in tumor growth, EMT, metastasis, and chemoresistance in brain cancers.

## Introduction

Brain cancers, as one of the most aggressive malignancies, cause high mortality in adults and children. In adults, meningioma, pituitary adenoma (PA), and glioblastoma (GBM) are common, but medulloblastoma is more common in children (Lapointe et al., 2018[20]; Reynoso-Noverón et al., 2021[42]). Surgical intervention, radiation, and chemotherapy are common therapeutic options for brain cancer. However, the median survival time cannot be improved by these therapeutic strategies and there is an urgent need to identify effective therapies to decrease mortality rates in cancer patients. Emerging evidence highlights the role of ncRNAs as master regulators involved in tumorigenesis and metastasis by stimulating the activity of various oncogenic signaling pathways like Wnt/β-catenin axis (Rahmani et al., 2018[34], 2021[36], 2022[37]; Soleimani et al., 2018[48]). Due to the crucial role of these oncogenic routes in tumor formation and development, promoting their activity is regarded as one of the main processes involved in human tumorigenesis. (Rahmani et al., 2020[40][33]; 2023[39]). 

Wnt signaling route is classified into β-catenin dependent (canonical) and β-catenin independent (noncanonical) pathways (Rahmani et al., 2018[34]). The canonical Wnt pathway was shown to have a critical impact on tumor growth, angiogenesis, invasion, and metastasis (Rahmani et al., 2019[32], 2020[40]; Amerizadeh et al., 2022[3]). In the activated form, the Wnt ligands were bound to their receptors including LDL receptor-related protein 5 or 6 (LRP-5/6) and Frizzled proteins resulted in the inactivation of β-catenin destruction complexes including glycogen synthase kinase 3β (GSK 3β), adenomatous polyposis coli (APC), casein kinase 1α (CK 1α), and Axin proteins. In this condition, the cytoplasmic β-catenin molecules are transferred to the nucleus and elevate transcription activity of T-cell factor/lymphocyte enhancer factor (TCF/LEF) complexes to increase expression of Wnt downstream effectors like survivin, c-Myc, matrix metalloproteinases, and cyclin D1 (Rahmani et al., 2018[34], 2020[40]). Cyclin D1 protein was shown to be upregulated in several tumors like GBM, hepatocellular carcinoma (HCC), colorectal cancer (CRC), and prostate cancer (PC) (Seifi et al., 2020[43]; Rahmani et al., 2021[36]).

Accumulating data suggest that several ncRNAs have essential roles in modulating the canonical Wnt axis which is over-activated in various types of human malignancies (Takao Real Karia et al., 2021[51]; Ji et al., 2022[17]; Rahmani et al., 2022[37]). Mechanistically, ncRNAs regulate Wnt/β-catenin route by suppressing Wnt downstream proteins like cyclin D1 and β-catenin suggesting a promising approach for better management and treatment of brain cancers.

## Role of ncRNAs in Tumorigenesis

Genome sequencing studies revealed that only about 2 % of the human genome is transcribed into a protein, and the rest of the RNA transcripts are considered ncRNAs including microRNAs (miRNAs), long non-coding RNAs (lncRNAs), and circular RNAs (circRNAs) (Khanbabaei et al., 2022[18]; Zeng et al., 2022[68]). miRNAs are endogenous small RNAs (18-23 nucleotides) that inhibit the expression of certain mRNAs by directly bound to their 3'-untranslated regions (UTRs) (Ali Syeda et al., 2020[2]). The role of miRNAs as oncogene or tumor suppressor genes in regulating various cellular processes including cell cycle progression, proliferation, and apoptosis, is widely recognized (Rahmani et al., 2020[40][38][35]). LncRNAs, as another group of ncRNAs with at least 200 nucleotides, were shown to be implicated in different biological processes by modulating the expression of their target genes implicated in cell proliferation, survival, and apoptosis by operating as a competing endogenous RNA (ceRNA) for certain miRNAs (Wang et al., 2019[57]; Basera et al., 2022[4]).

CircRNAs are known as a stable group of ncRNAs that are more resistant to nuclease enzymes due to their cyclic structure. Like lncRNAs, circRNAs function as ceRNA for certain miRNAs and regulate their expression at various transcriptional levels (Aboutalebi et al., 2020[1]). Emerging evidence suggests that uncontrolled expression of ncRNAs facilitates tumor growth and progression through stimulating cellular signalings involved in proliferation and metastasis (Lv and Huang, 2019[27]; Rahmani et al., 2022[37]).

## The Interplay between ncRNAs and Wnt/β-catenin Pathway in Glioblastoma

Glioblastoma (GBM) is among the most common malignancies in the CNS with a dismal survival rate (Si et al., 2020[45]; Stylli, 2020[49]; Payazdan et al., 2021[31]). GBM tumorigenesis is a complicated process that involves multiple genetic modifications like upregulation of oncogenes or suppression of tumor suppressors that stimulate the activity of different signaling pathways including Wnt/β-catenin axis (Zuccarini et al., 2018[73]; Latour et al., 2021[21]). 

Recent studies on the molecular mechanisms involved in human tumorigenesis indicate that various lncRNAs promote GBM growth and progression by inhibiting certain anti-cancer miRNAs including miR-15a/b-5p and miR-5095 (Ren et al., 2018[41]; Zheng et al., 2019[68]; Li et al., 2020[22]; Wang et al., 2021[59]). Consistently, increased expression of lncRNA AGAP2 antisense RNA 1 (AGAP2-AS1) is related to poor prognosis and lower survival rates in GBM patients. It has been found that AGAP2-AS1 is critically implicated in GBM development and progression by targeting miR-15a/b-5p and upregulation of β-catenin and cyclinD1 in the canonical Wnt axis (Zheng et al., 2019[68]).

Likewise, the lncRNA DLGAP1-AS1 as a ceRNA for miR-515-5p upregulates the Rho-associated coiled-coil containing protein kinase 1 (ROCK1) and enhances tumor growth by inducing the canonical Wnt pathway (Wang et al., 2021[59]). ROCK1 as one of the main components of Wnt signaling was reported to induce tumor cell invasion and metastasis in various cancers including non-small cell lung cancer (NSCLC), breast cancer, and GBM (Maskey et al., 2017[30]; Ko et al., 2021[19]). Therefore, the DLGAP1-AS1/miR-515-5p/ROCK1 route may be investigated as a promising therapeutic axis for GBM.

LncRNA small nucleolar RNA host gene 7 (SNHG7) is another tumor-related lncRNA that induces GBM tumorigenesis by sponging miR-5095. The expression of SNHG7 is observed to be inversely associated with clinical outcomes and survival rate in patients with GBM (Ren et al., 2018[41]). Restoration of SNHG7 facilitates cancer progression and metastasis by inhibiting miR-5095 and upregulating β-catenin, cyclinD1, and c-myc proteins (Ren et al., 2018[41]). In addition, upregulation of lncRNA small nucleolar RNA host gene 17 (SNHG17) is observed in various cancers including NSCLC, gastric cancer (GC), and GBM. SNHG17 located in chromosome 20q11.23 has critical effects on regulating tumor cell growth and apoptosis. Li et al. illustrated that SNHG17 suppresses miR-506-3p to enhance the expression of CTNNB1/β-catenin and elevates GBM cell invasion and metastasis (Li et al., 2020[22]). The expression of CTNNB1 was also shown to be upregulated by lncRNA SNHG5. It has been shown that the knockdown of SNHG5 inhibits malignant features of GBM by suppressing CTNNB1 and downregulating Wnt/β-catenin signaling axis (Chen et al., 2019[6]). 

As mentioned before, the β-catenin protein as the main effector of canonical Wnt signaling has critical effects in regulating cell proliferation, migration, and metastasis. β-catenin forms activating complexes with the nuclear transcription factors and upregulates cyclinD1 and c-myc (Rahmani et al., 2020[38][35]). As presented in Table 1(Tab. 1) (References in Table 1: Beylerli et al., 2020[5]; Chen et al., 2019[6]; Dhanyamraju et al., 2020[8]; Dong et al., 2017[9]; Fu et al., 2018[11]; Ghafouri-Fard et al., 2021[12][13]; Ko et al., 2021[19]; Li et al., 2018[23], 2019[24][25]; Lu et al., 2020[26]; Ma et al., 2022[28]; Mao et al., 2022[29]; Ren et al., 2018[41]; Shen et al., 2020[44]; Smitha et al., 2021[46]; Soleimani et al., 2019[47]; Sun et al., 2020[50]; Wang et al., 2018[53], 2021[59]; Zhang et al., 2020[63]; Zhao et al., 2021[67]; Zheng et al, 2019[68]; Zhou et al., 2022[70]; Zuccarini et al. 2018[73]), there are multiple oncogenic lncRNAs that induce the expression of β-catenin in GBM.

For example, the expression of lncRNA HOXA13 is increased in several cancers, including HCC, PC, and GBM, which is associated with dismal prognosis and aggressive features in cancer patients (Dong et al., 2017[9]). Duan et al. showed that HOXA13 promotes GBM progression and metastasis by upregulating β-catenin and inducing the canonical Wnt pathway (Duan et al., 2015[10]). More importantly, it has been shown that HOXA13 induces tumor cell metastasis by regulating the expression of SMAD 2 and SMAD3 (Duan et al., 2015[10]). The SMAD proteins function as transcription factors in the TGF pathway and have crucial effects in promoting tumor invasion and EMT (Soleimani et al., 2019[47]). Similarly, the overexpression of lncRNA bladder cancer-associated transcript 1 (BLACAT1) is reported to be associated with tumor malignant features in patients with GBM. Increased expression of lncRNA BLACAT1 is reported in several cancers including GBM, NSCLC, GC, and cervical cancer (CC) (Wang et al., 2018[53]; Li et al., 2019[25]). Silencing BLACAT1 reduces cancer growth, EMT, and metastasis by regulating β-catenin, cyclin D1, vimentin, and cadherin proteins (Li et al., 2019[25]). Moreover, the expression of oncogenic lncRNA ADAMTS9-AS1 was observed to be correlated with tumor growth and aggressiveness in patients with GBM. LncRNA ADAMTS9-AS1 located on chromosome 3p14.1 is implicated in cancer cell proliferation, invasion, and metastasis. Zhou et al. reported that depletion of lncRNA ADAMTS9-AS1 inhibits GBM progression and metastasis by modulating β-catenin, c-myc, and E-cadherin (Zhou et al., 2022[70]). Similarly, lncRNA DANCR as another oncogenic ncRNA was reported to promote GBM metastasis by upregulating β-catenin, c-myc, and vimentin, and suppressing E-cadherin (Li and Zhou, 2018[23]). 

β-catenin was also shown to be upregulated by LINC01503. Upregulation of lncRNA LINC01503 is associated with GBM progression and aggressiveness. Downregulation of LINC01503 inhibits GBM metastasis while induces apoptosis through suppressing β-catenin and cyclinD1 (Wang et al., 2019[55]). Similarly, downregulation of lncRNA H19 inhibits cell cycle progression, proliferation, and metastasis by downregulating β-catenin and cyclin D1 (Guan et al., 2019[16]). 

Among all lncRNAs which induce GBM tumorigenesis, there are multiple tumor-suppressor lncRNAs that suppress GBM by inhibiting the Wnt/β-catenin route (Table 2(Tab. 2); References in Table 2: Derderian et al., 2019[7]; Gong et al., 2019[15]; Guan et al., 2019[16]; Tian et al., 2019[52]; Wang et al., 2019[57]; Wu et al., 2021[60]; Zhang et al., 2019[64]; Zhu et al., 2020[71]). For example, decreased expression of lncRNA GAS8-AS1 has been observed in various tumors like thyroid carcinoma, osteosarcoma, CRC, and GBM. Upregulation of GAS8-AS1 reduces GBM cell proliferation, and metastasis by downregulating β-catenin, cyclinD1, and axin2 (Wu et al., 2021[60]). Similarly, tumor-suppressive lncRNA cancer susceptibility candidate 7 (CASC7) has been shown to inhibit GBM tumorigenesis by suppressing β-catenin and cyclinD1 (Gong et al., 2019[15]). The lncRNA Linc00320 is another tumor-suppressive lncRNA whose reduced expression is correlated with cancer growth and progression in GBM patients. Tian et al. demonstrated that the lncRNA Linc00320 suppresses Wnt/β-catenin axis by disrupting the β-catenin/TCF4 complex in GBM cells (Tian et al., 2019[52]). 

Moreover, the lncRNA LINC p53 induced transcript (LINC-PINT) has been shown to function as an anti-cancer ncRNA in several cancers like GBM, CRC, NSCLC, esophageal cancer, and melanoma (Zhang et al., 2019[64]; Zhu et al., 2021[72]). Zou et al. demonstrate that LINC-PINT inhibits tumor invasion and metastasis by modulating β-catenin and vimentin (Zhu et al., 2021[72]). 

## The Interplay between ncRNAs and Wnt/β-catenin Pathway in Pituitary Cancer

Pituitary adenoma (PA) is the third most frequent brain cancer after meningioma and GBM. Recent findings indicated the oncogenic role of ncRNAs in PA tumorigenesis by stimulating the canonical Wnt signaling (Beylerli et al., 2020[5]; Xue and Ge, 2020[61]). For instance, aberrant expression of lncRNA colon cancer-associated transcript 2 (CCAT2) is associated with tumor development and metastasis in multiple human malignancies, including PA, HCC, CRC, and breast cancer (Fu et al., 2018[11]). In support of the oncogenic function of CCAT2, Fu et al. demonstrated a significant G1/S arrest and apoptosis in CCAT2-silenced cells whereas upregulation of CCAT2 induces cell cycle progression and reduces apoptosis (Fu et al., 2018[11]). *In vitro* experiments illustrate that this lncRNA induces PA progression and invasiveness by regulating MMP2 and 13 (Fu et al., 2018[11]; Ma et al., 2022[28]).

Further investigations demonstrated that some lncRNAs can induce PA tumorigenesis by silencing several tumor-suppressor miRNAs at transcriptional level. The overexpression of lncRNA prostate cancer-associated transcript6 (PCAT6) has been reported in many malignant tumors like PA, NSCLC, PC, CC, and OC (Ghafouri-Fard et al., 2021[13]). Zao et al. demonstrated that PCAT6 regulates miR-139-3p in modulating cell cycle progression, apoptosis, migration, and invasion (Zhao et al., 2021[67]). Mechanism research has shown that PCAT6 promotes cancer growth by sponging tumor suppressor miR-139-3p that downregulates Bcl-2 and BRD4 while induces Bax and Cleaved caspase-3. Moreover, PCAT6 induces tumor metastasis by directly regulating EMT-related proteins like E-cadherin and N-cadherin (Zhao et al., 2021[67]).

Consistently, Mao et al. reported that the lncRNA small nucleolar RNA host gene 6 (SNHG6) accelerates EMT and metastasis by targeting miR-944 (Shen et al., 2020[44]). The tumor suppressive miR-944 was reported to reduce PA growth and progression by regulating the expression of E-cadherin and vimentin. To explore the oncogenic mechanism of SNHG6 in pituitary tumors, it has been shown that SNHG6 upregulates vimentin but inhibits E-cadherin expression. These findings indicate that the lncRNA SNHG6, as a ceRNA for miR-944, induces pituitary cancer invasion and metastasis by regulating EMT-related proteins (Mao et al., 2022[29]). 

Increased expression of plasmacytoma variant translocation 1 (PVT1) is also reported in a number of cancers like PA, nasopharyngeal carcinoma, and renal cancer (Derderian et al., 2019[7]). Ectopic expression of PVT1 potentially induces cell growth, chemo-resistance, migration, and EMT while silencing its expression decreases PA cell proliferation and downregulates β-catenin and cyclin D1 (Zhang et al., 2019[65]).

In addition to the oncogenic lncRNAs, there are multiple tumor-suppressor lncRNAs that reduce PA carcinogenesis by suppressing canonical WNT pathway. For instance, downregulation of lncRNA CLRN1-AS1 in PA is shown to be negatively correlated with cancer progression and aggressiveness (Wang et al., 2019[54]). Mechanistically, lncRNA CLRN1-AS1 upregulates the dickkopf WNT inhibitor 1 (DKK1) by targeting miR-217. Elevated expression of CLRN1-AS1 alleviates cancer progression by repressing the canonical Wnt signaling and downregulating cyclin D1, c-myc, beclin, and β-catenin. In addition, it has been shown that CLRN1-AS1 induces PA cell apoptosis by regulating the activity of caspase3 (Wang et al., 2019[54]). 

Decreased expression of maternally expressed 3 (MEG3) is associated with tumor development and metastasis in PA (Zhu et al., 2020[71]). Ectopic expression of MEG3 attenuated tumor EMT and invasion by influencing the expression of MMP7, E-cadherin, and survivin. Further studies on the role of MEG3 on tumor cell apoptosis indicate that MEG3 sponges miR-23b-3p and restores the expression of FOXO4. It has been shown that FOXO4 promotes apoptosis and cell cycle arrest by regulating Bcl-xl, Bcl-6, and Bcl-2. Taken together, these findings clearly support the anti-tumor activity of MEG3 and present a novel target for PA treatment (Wang et al., 2021[58]). 

Moreover, decreased expression of growth arrest specific transcript 5 (GAS5) in PA induces tumor aggressive behavior and metastasis. It has been reported that the lncRNA GAS5 suppresses miR-27a-5p and elevates the expression of cylindromatosis (CYLD) in tumor cells, resulting in decreased cell proliferation and tumor growth (Wang et al., 2022[56]). Tables 1(Tab. 1) and 2(Tab. 2) summarize the role of lncRNAs in PA.

## The Interplay between ncRNAs and Wnt/β-catenin Pathway in Meningioma

Meningioma is the second type of CNS cancer with an incidence of about 40 % (Smitha and Sivaraman, 2021[46]). The current treatment regimen is not fully effective and the prognosis of patients with meningioma is not favorable. Thus, there is a great need for discovering novel therapeutic methods for meningioma. To identify novel therapeutic targets for meningioma, various lncRNAs are identified that induce tumorigenesis by targeting various anti-cancer miRNAs and modulating their target genes (Ghafouri-Fard et al., 2021[12]).

For example, the elevated expression of small nucleolar RNA host gene 1 (SNHG1) in various tumors like GC, CRC, GBM, and meningioma, is related to tumor growth and development (Zhang et al., 2020[66]). SNHG1 induces canonical Wnt signaling by negatively regulating miR-556‐5p. To investigate the anti-cancer mechanism of miR-556-5p, it has been found that this miRNA attenuates cancer cell proliferation by targeting Wnt signaling-related transcription factors including TCF12. Altogether, these findings indicate that regulating the SNHG1/miR‐556‐5p/TCF12 axis may have therapeutic potential for meningioma (Zhang et al., 2020[66]).

The long intergenic non-protein coding RNA 702 (LINC00702) is another cancer-related lncRNA whose expression is related to poor prognosis and malignant growth in meningioma (Li et al., 2019[24]). In mechanism, LINC00702 activates the canonical Wnt pathway by sponging miR-4652-3p and upregulating the transcription factor ZEB1. Li et al. showed that silencing LINC00702 efficiently decreases tumor cell viability and migration via suppressing β-catenin and c-myc (Li et al., 2019[24]). Their findings indicate that LINC00702 exerts its oncogenic effects by modulating the miR-4652-3p/ZEB1 axis and upregulating the canonical Wnt pathway (Li et al., 2019[24]). Table 1(Tab. 1) demonstrates the role of lncRNAs in meningiomas.

## The Interplay between ncRNAs and Wnt/β-catenin Route in Medulloblastoma

Medulloblastoma is the most common cancer of CNS in children with high metastasis and poor survival rate (Dhanyamraju et al., 2020[8]). Recent data indicate that various lncRNAs induce medulloblastoma tumorigenesis by facilitating tumor migration, EMT, and invasiveness. It has been reported that upregulation of HOX transcript antisense RNA (HOTAIR) associated with cancer growth and development in medulloblastoma (Zhang et al., 2020[63]). Enforced expression of HOTAIR significantly accelerates tumor progression and development in medulloblastoma through targeting miR-1 and miR-206 and inducing Yin Yang 1 (YY1) transcription factor. Recent studies revealed that YY1 induces human carcinogenesis by regulating various oncogenic routes like PI3K/AKT and Wnt pathway. Therefore, the HOTIAR-miR-1/miR-206-YY1 axis can be investigated as a novel molecular target with therapeutic potential for medulloblastoma (Zhang et al., 2020[63]). 

In another report, the anti-cancer miR-29c-3p is shown to be downregulated by the lncRNA Colorectal neoplasia differentially expressed (CRNDE) in medulloblastoma (Sun et al., 2020[50]). Enhanced expression of miR-29c-3p inhibits cancer cell growth, and metastasis, but induces sensitivity to cisplatin. To investigate the tumor-related function of CRNDE, Sun et al. illustrate that downregulation of CRNDE inhibits tumor aggressiveness by suppressing miR-29c-3p and inhibiting the canonical Wnt pathway (Sun et al., 2020[50]). Increased expression of CRNDE has been reported in many cancers which is associated with chemoresistance, and adverse clinical outcomes (Lu et al., 2020[26]). 

Urothelial carcinoma associated 1 (UCA1) is another oncogenic lncRNA whose expression is positively correlated with tumor invasiveness and adverse clinical outcomes in medulloblastoma (Zhengyuan et al., 2017[69]). Upregulation of UCA1 was reported in multiple cancers, including GBM, HCC, NSCLC, GC, and, bladder cancer (Ghafouri-Fard and Taheri, 2019[14]). The oncogenic effects of UCA1 in human tumors were mediated by recruiting Wnt ligands and inducing Wnt signaling. To further investigate the oncogenic mechanism of UCA1, Zhengyuan et al. demonstrated that the knockdown of UCA1 reduces cell cycle progression, proliferation, and migration in medulloblastoma cells and tissues (Zhengyuan et al., 2017[69]). Table 1(Tab. 1) demonstrates the role of lncRNA in medulloblastoma.

## Conclusion

**Figure 1:**
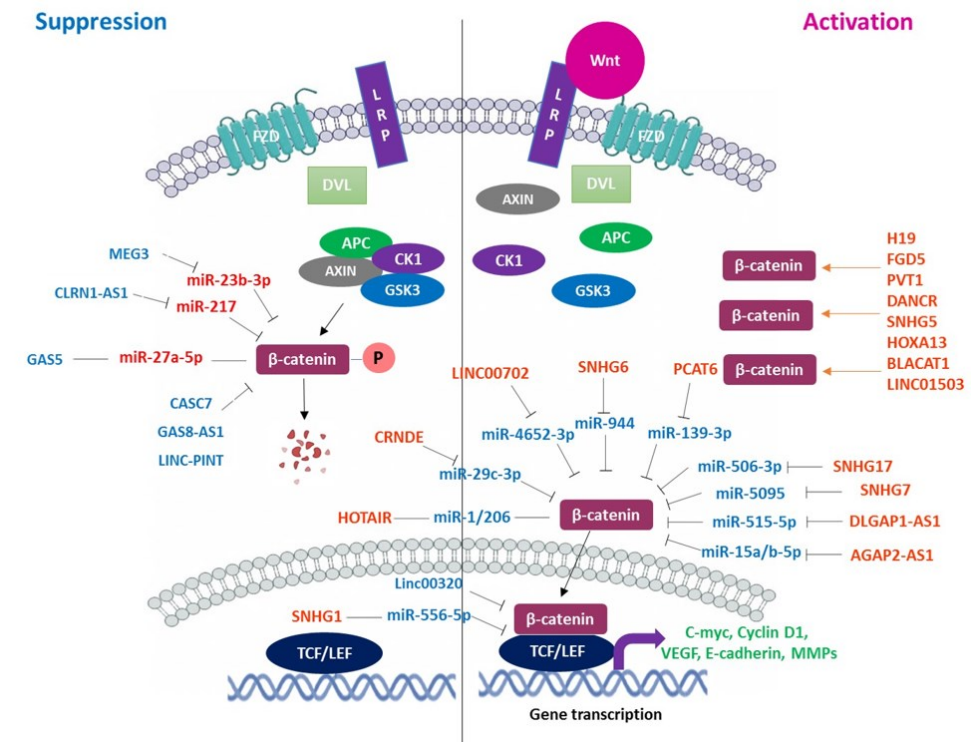
Schematic representation of regulatory effects of ncRNAs on the activity of the Wnt/β-catenin signaling contributed to the pathogenesis of brain cancers

In this study, we presented recent findings about the role of ncRNAs in brain cancer tumorigenesis through regulating canonical Wnt signaling. As is shown, the Wnt/β-catenin axis has critical effects on cell growth, survival, and proliferation. Recent findings indicate that ncRNAs have potent regulatory effects on the Wnt pathway resulting in tumor initiation and progression (Table 1(Tab. 1) and 2(Tab. 2)) (Zhengyuan et al., 2017[69]). As presented in Figure 1(Fig. 1), many Wnt pathway-related ncRNAs are aberrantly expressed in human brain cancers, which may function as an oncogene or tumor suppressor. These ncRNAs can promote or inhibit the canonical Wnt signaling by regulating GSK3, APC, β-catenin, and TCF/LEF transcription factors resulting in modulating several Wnt downstream targets involved in cell proliferation, invasion, EMT, and metastasis. Therefore, treatments targeting the Wnt signaling-related ncRNAs may have therapeutic potential for improving quality of life and increasing overall survival rates in brain cancer patients.

## Notes

Farzad Rahmani and Seyed Mahdi Hassanian (Basic Medical Sciences Institute, Mashhad University of Medical Sciences, Mashhad, Iran; E-mail: Hasanianmehrm@mums.ac.ir) contributed equally as corresponding author.

## Declaration

### Author contributions 

FR and AMA wrote the manuscript. RH, MZ and HF contributed to the final manuscript. MP and PS verified and discussed the studies. MR edited and proofread the manuscript. SMH supervised the study.

### Funding

This study was supported by grants awarded by the Mashhad University of Medical Sciences (Grant No. 990306) to Farzad Rahmani.

### Conflict of interest

The authors declare that they have no conflict of interest.

## Figures and Tables

**Table 1:**
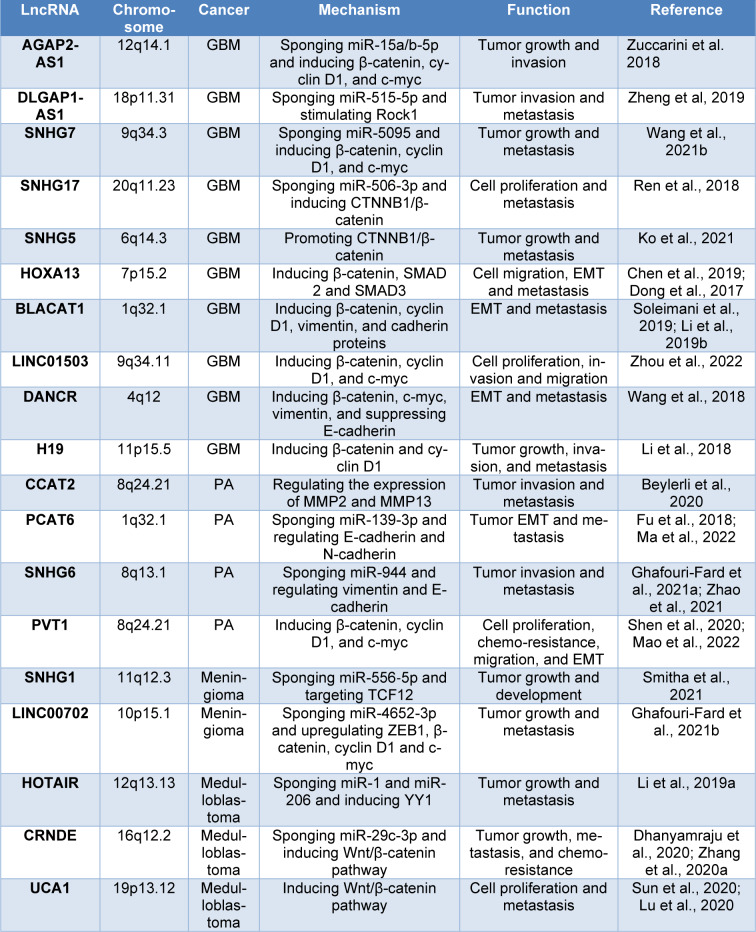
Oncogenic lncRNAs

**Table 2:**
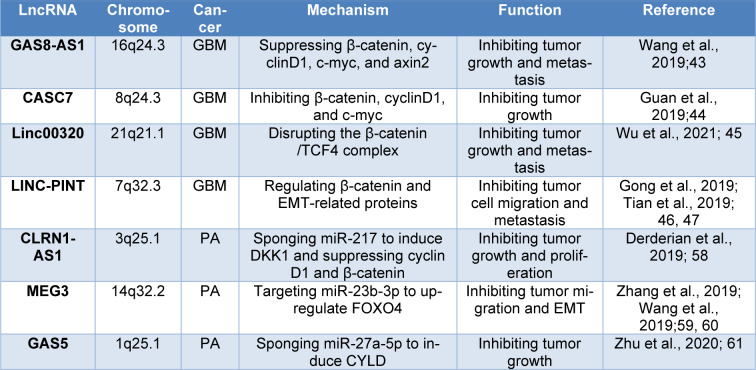
Tumor suppressive lncRNAs
